# Retraction: Circular ribonucleic acid circFTO promotes angiogenesis and impairs blood–retinal barrier *via* targeting the miR-128-3p/Thioredoxin interacting protein axis in diabetic retinopathy

**DOI:** 10.3389/fmolb.2025.1727243

**Published:** 2025-11-18

**Authors:** Frontiers Editorial Office

The journal retracts the 4 August 2021 article cited above.

Following publication, concerns were raised regarding the integrity of the data in the published Figure 6D, and the subsequent duplication 5 months later of this image in another journal’s published article (Bioengineered, 2021 December; 12 (2):10187–10198. doi: 10.1080/21655979.2021.2001217). The authors failed to provide the raw data or a satisfactory explanation during the investigation, which was conducted in accordance with Frontiers’ policies. As a result, the data and conclusions of the article have been deemed unreliable and the article has been retracted.

This retraction was approved by the Field Chief Editor of Frontiers in Molecular Bioscience and the Chief Executive Editor of Frontiers. The authors have not responded to correspondence regarding this retraction.

Frontiers would like to thank Rene Aquarius for contacting the journal regarding the published article.

